# Congenital tuberculosis in preterm infants in a high-burden setting in southwest China: a single-center cross-sectional study

**DOI:** 10.3389/fped.2025.1657411

**Published:** 2025-11-04

**Authors:** Xiaoling Zhuang, Wanting Xu, Wen Xu, Yan Shi, Mei Xiao, Awai Rihei, Wenbin Dong, Chengpeng Bian

**Affiliations:** ^1^ Department of Pediatrics, The First People’s Hospital of Liangshan Yi Autonomous Prefecture, Liangshan, China; ^2^ Department of Neonatology, Children’s Medical Center, The Affiliated Hospital of Southwest Medical University, Luzhou, Sichuan, China

**Keywords:** premature infants, congenital tuberculosis, assisted reproduction, clinical features, laboratory examinations

## Abstract

**Background:**

Congenital tuberculosis (CTB) is an extremely rare and potentially life-threatening infection in premature infants that is frequently misdiagnosed. There is still a lack of thorough characterization of this condition in preterm neonates, particularly in high TB-burden settings.

**Methods:**

Premature infants with CTB were identified from hospital medical records from January 2016 to December 2023 in a high-burden, resource-limited neonatal intensive care unit (NICU) in southwest China. Diagnosis was based on etiology and clinical evidence. Data extracted included demographics, maternal history of tuberculosis (TB) exposure, symptoms, laboratory markers, microbiological findings, imaging findings, treatment regimens, and prognosis. These were evaluated during the pre-diagnosis, diagnosis, and posttreatment stages.

**Results:**

A total of 11 premature infants with CTB were included. Maternal TB was common in this cohort; 7 of the 11 mothers were diagnosed with TB following *in vitro* fertilization and embryo transfer. Clinical manifestations were atypical and consisted primarily of non-specific symptoms, including low fever, shortness of breath, poor reaction, less eating, and coughing. Laboratory findings during the active phase of CTB revealed statistically significant elevations in C-reactive protein levels (*P* = 0.001), thrombocytopenia (*P* = 0.007), hyponatremia (*P* = 0.040), hypocalcemia (*P* = 0.022), and hypomagnesemia (*P* = 0.025). Sputum acid-fast bacillus (AFB) smear was positive in 4 out of 11, while gastric juice AFB smear was positive in 6 out of 11. Mycobacterial liquid culture produced the highest positivity with 7 out of 11, followed by solid culture and interferon-gamma release assays with 5 out of 11. All nucleic acid amplification tests were positive, and chest CT scans showed abnormalities in each patient*.* Among the patients, five experienced liver function impairment after anti-TB treatment, as evidenced by elevated alanine aminotransferase levels.

**Conclusions:**

In this study, preterm infants with CTB frequently demonstrated non-specific clinical signs with a reversible pattern of inflammation, anemia, thrombocytopenia, and electrolyte disturbances that normalized after anti-TB therapy. These patterns, together with maternal TB risk or *in vitro* fertilization and embryo transfer history, may raise clinical suspicion and justify the early use of nucleic acid amplification testing, and generalizability outside this setting requires larger, controlled cohorts.

## 1 Introduction

Tuberculosis (TB) continues to be a significant global health challenge, with an estimated 10.6 million new cases and 1.3 million deaths reported in recent years. In recent years, the incidence of TB has increased by 3.9%, reversing previous declines despite efforts to control the disease (1). Congenital tuberculosis (CTB), due to *Mycobacterium tuberculosis* (MTB) transmission *in utero* or during delivery, is still a very uncommon form of TB in newborns, making up <1%–2% of all pediatric TB cases worldwide. Non-specific clinical presentations, such as respiratory distress, fever, and hepatosplenomegaly, which can be mistaken for neonatal sepsis or pneumonia, make diagnosis difficult and often cause delays in starting the right treatment. Misdiagnosis rates have been reported as high as 59.78% (2). Although extremely rare, the fatality rate of CTB is nearly 100% if left untreated, and remains between 20% and 50% even with current anti-TB treatments (3). Despite being a particularly vulnerable subgroup due to their immature immune systems and frequent complications such as respiratory distress syndrome (RDS) and bronchopulmonary dysplasia (BPD), there are few systematic data on CTB in preterm neonates.

Southwest China was selected as a high-burden setting due to its unique geographical environment, economic conditions, and population mobility contributing to TB (4). Located in Sichuan Province, southwest of China, Liangshan Yi Autonomous Prefecture is distinguished by increased etiological positive rates of TB in children, widespread poverty, and substantial obstacles to accessing healthcare (5). Although maternal TB is found in >90% cases with CTB (6), there is little epidemiological information specifically addressing the relationship between endemic CTB, prematurity, and outcomes in areas with high rates of burden and limited resources, particularly in NICUs in southwest China. The majority of what is currently known about preterm infants and the regional sociodemographic factors influencing outcomes is based on isolated case reports, which frequently lack details of a case retrospective study (7–10).

In addition, the First People's Hospital of Liangshan Yi Autonomous Prefecture served as the largest infectious disease hospital in Liangshan Yi Autonomous Prefecture, as well as a regional referral center, and was recognized as a high-burden area of TB (11). Clinical data of premature infants with CTB from January 2016 to December 2023 in a high-burden, resource-limited neonatal intensive care unit (NICU) in southwest China were retrospectively analyzed to describe clinical and laboratory patterns in a preterm cohort with CTB, identify pragmatic triage signals and early treatment strategies, and reduce mortality. This study also analyzed the observing patterns, diagnostic protocols, laboratory examinations, treatments, and prognosis of 11 premature infants with CTB.

## 2 Materials and methods

### 2.1 Study design and data collection

This cross-sectional study was conducted at the First People's Hospital of Liangshan Yi Autonomous Prefecture, China. The infants were hospitalized in the neonatal department between January 2016 and December 2023, and laboratory analyses were performed at the local Tuberculosis Prevention and Control Institute. The medical records of all infants were independently reviewed by three senior physicians to prevent missed diagnoses.

CTB was defined as follows: the mother's MTB infection of the placenta, the umbilical cord blood, or the amniotic fluid could be inhaled or swallowed by the fetus. In a small percentage of pregnant women with genital TB, the fetus may inhale or swallow MTB during the delivery process, which could cause neonatal illness.

CTB was diagnosed based on the criteria put forward by Cantwell et al. (12) in 1994, which included four types: (1) TB within 1 week after birth; (2) primary hepatic complex or intrahepatic caseous granuloma; (3) placental or maternal genital TB infection; and (4) exclusion of postnatal TB infection. Diagnosis also followed current guidelines for treating infants exposed to TB, including the neonatal hospital staff.

The following were the inclusion requirements of this research: (1) gestational age under 37 weeks; (2) symptoms of infection poisoning, including fever, coughing, shortness of breath, apnea, poor reaction, and decreased appetite [increased C-reactive protein (CRP) and thrombocytopenia as primary indicators of infection]; (3) poor efficacy of broad-spectrum antibiotics and antifungal therapy; (4) one or more positive in auxiliary examinations: sputum and gastric juice acid-fast bacillus (AFB) smear, sputum and gastric juice MTB gene Xpert detection and drug resistance gene test [Xpert MTB/rifampicin (RIF)], and blood MTB infection T lymphocyte spot test (spot test of MTB infection T lymphocytes, T-SPOT.TB); and (5) chest X-ray revealing changes in exudation, millet shadow, or patchy shadow.

The following were the exclusion criteria: (1) gestational age above 37 weeks; (2) lack of etiological test results for children suspected of TB infection; and (3) neonatal with TB infection after birth through epidemiological investigation.

A disposable airtight sputum sample collection device was used to draw sputum samples from secretions under negative pressure through the oropharynx or glottis. At the same time, an indwelling gastric tube in the premature infants was used to collect fasting gastric juice of 3–5 mL in the early morning. In addition, gastric aspirates and sputum samples were collected within the first 48 h of suspicion for 3 consecutive days. Both sputum and gastric juice samples were collected in clearly labeled, wide-mouthed, transparent, and leak-proof containers and were sent to the TB institute for further examination. Blood samples of the tuberculin sensitivity test (TST) were also collected. Chest images were reviewed independently by two pediatric radiologists blinded to the clinical information. The findings were assessed using a standardized qualitative scoring system adapted for pediatric TB (13), evaluating the following domains: parenchymal involvement, including consolidation, nodules, and miliary pattern. Lymph node enlargement involved a short-axis diameter of ≥10 mm. Pleural disease and sequelae represented atelectasis or fibrosis.

General information on the newborn and the mother was collected by examining medical records, including the method of gestation, previous pregnancies and histories of pregnancies, whether the mother had a history of TB, and what type of TB. Sex, gestational age, method of delivery, age of onset, age of diagnosis, clinical signs, auxiliary examination and treatment of TB, outcome, follow-up, and prognosis were also documented. To minimize missing data and inconsistencies, the primary investigator and supervisors of the research closely monitored the data collection procedure. No identifiable or personal information was gathered for this research.

### 2.2 Laboratory examination

In our cohort study, T-SPOT.TB assays were conducted using the commercial ELISPOT T-SPOT.TB kit (Oxford Immunotec, UK) according to the manufacturer's instructions. Two antigen panels were used to activate antigen-specific T cells (Panel A, ESAT-6; Panel B, CFP-10). Using the following criteria, the results were interpreted according to the number of spot-forming units (SFUs). Positive results were defined as at least eight SFUs more than the negative control in either ESAT-6 or CFP-10 wells. Negative results were defined as fewer than five SFUs than the negative control in both ESAT-6 and CFP-10 wells. Indeterminate results indicated a failure of the positive control or a high level of background for the negative control. N-acetyl L-cysteine (NALC) NaOH was used to process the respiratory samples before they were further processed for Xpert testing, smear, and culture. Auramine and Gram stains were used to stain the smears, which were then examined under light and fluorescent microscopy, respectively, to examine other bacterial pathogens and acid-fast MTB bacilli. Xpert MTB/RIF results, including semiquantitative categories, were generated by the instrument’s integrated software using its inherent classification system. In accordance with WHO guidelines, acid-fast bacilli were graded on smears (14). Both liquid culture of mycobacterial growth indicator tube (MGIT) and solid Lowenstein–Jensen (LJ) culture were applied to the processed samples. In addition, MTB and its resistance to RIF were identified through Xpert MTB/RIF testing.

### 2.3 DNA extraction

The processed sample was combined with 30 µL of 10% sodium dodecyl sulfonate (SDS) and 3 µL of 20 µg/mL proteinase K and was then incubated at 56 °C for 1 h. After adding 100 µL of 5 M NaCl and 80 µL of CTAB/NaCl solutions, the mixture was incubated at 65 °C for 10 min. As a whole, DNA was extracted from each sample using the cetyltrimethylammonium bromide (CTAB) method (15). After adding an equal amount of chloroform and centrifuging at a speed of 10,000 rpm for 1 min, the supernatant was transferred. For the multiplex real-time polymerase chain reaction (RT-PCR), the supernatant was utilized.

### 2.4 Multiplex RT-qPCR

Multiplex real-time PCR was done using the QIAGEN Microbial DNA qPCR Array Kit for the detection of a panel of MTB pathogens on extracted DNA. Probes A–E are five molecular beacons that target the 81 bp region of the rpoB gene that determines rifampin resistance. Following the kit's instructions, the real-time PCR results were interpreted. Sample processing control (SPC) acts as an internal reference for sample processing. Quality control (QC) serves as the quality control within the system. A *C*_t_ value between 15.0 and 18.0 indicates a high bacterial load, suggesting that the microorganism may be a pathogen rather than a commensal. A *C*_t_ value of 18.1–23.0 indicates a medium load, while a value of 23.1–33.0 indicates a low load, suggesting commensal rather than pathogenic activity. The cited *C*_t_ value threshold is only applicable to the research-use-only multiplex RT-PCR assay used for supplementary rpoB targeting, not to the clinically reported Xpert MTB/RIF results. Moreover, each sample is repeated three times.

### 2.5 Treatment plan of CTB

Respiratory support and short-term empirical antibiotic treatment for episodes of suspected, culture-negative sepsis consisted of the initial management of these 11 premature infants with CTB. Among them, five received invasive mechanical ventilation and six received non-invasive mechanical ventilation. Blood analysis, serum biochemical and electrolyte indicators, chest x-ray, and cerebrospinal fluid (CSF) examinations were conducted before antibiotic treatment. All of them were grouped as pre-diagnosis group, whose samples were taken during the initial hospital stay, prior to confirmatory diagnostic testing, such as TB culturing, or multiplex RT-PCR, when clinical symptoms suggestive of CTB were present. This typically occurred within 24–48 h of admission. The diagnosis group was defined as samples taken on the day of or the day after a confirmed diagnosis of CTB, at the time of confirming the microbiological or histopathological results were positive. Posttreatment group included samples taken following the conclusion of a typical course of anti-TB treatment, which usually lasted for 9 months. Follow-up samplings, including examinations of blood indicators, serum biochemical indicators, and electrolyte indicators, were performed during outpatient visits within 2 weeks after treatment completion.

**Figure 1:**
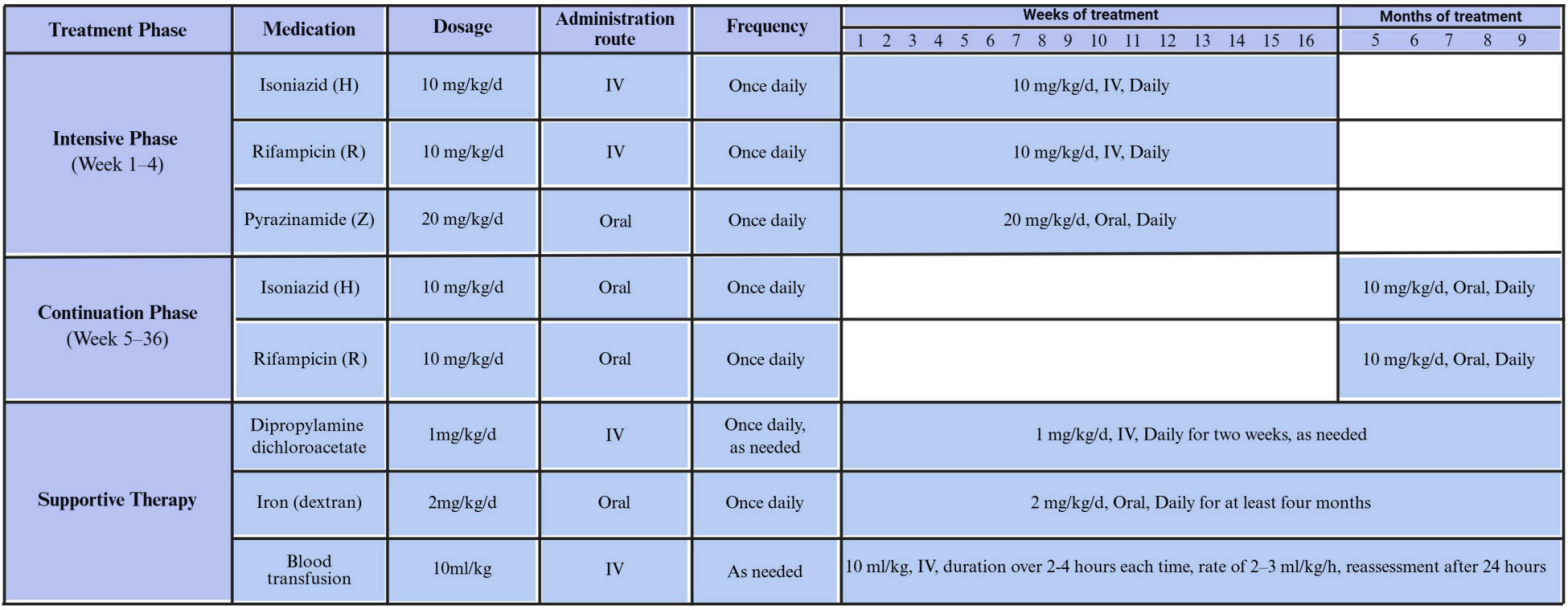
The treatment plan for CTB in premature infants. CTB, congenital tuberculosis.

The treatment plan for CTB in premature infants is (3H-R-Z-/9H-R). During the first 3 weeks, isoniazid and rifamycin are administered intravenously, combined with oral pyrazinamide. Isoniazid and rifampicin are administered intravenously at the low dosage of 10 mg/kg/d, and pyrazinamide is orally taken at the dosage of 20 mg/kg/d per day. The treatment is switched to all oral administration once the infant's condition improves. Hepatotoxicity is managed with supportive therapy using compound *diisopropylamine dichloroacetate*. Severe anemia infants received multiple blood transfusions and were given oral administration of dextran iron (Figure 1).

The following criteria are used to assess the effectiveness of treatment: improvement of the premature infant's clinical symptoms and signs, serum biochemical analysis, blood examinations of white blood cell (WBC) and hemoglobin counts, chest imaging changes, and other indicators; the results are classified as cure, improvement, or death (16).

The following criteria are used to diagnose liver injury after anti-TB treatment (17): (1) mild, which is typically reversible and only involves an increase in serum enzymes, and (2) moderate, which involves more extensive damage and early liver function impairment as indicated by elevated serum total bilirubin or other serious clinical illness with noticeable jaundice.

### 2.6 Statistical analysis

Data were analyzed by SPSS-AU. The significance level was recorded as 5%. The Kolmogorov–Smirnov test was applied to demonstrate whether blood analysis indicators, biochemical indicators, electrolyte indicators, and hemoglobin were normally distributed before analysis. Numerical data were indicated as the median (minimum-maximum) means ± standard deviations (SDs). Pairwise comparisons of the differences among the three groups, including the pre-diagnosis group, diagnosis group, and posttreatment group, were conducted using Tukey–Kramer tests. For data that were not normally distributed, the Wilcoxon signed-rank test was employed.

## 3 Results

### 3.1 Basic characteristics of preterm infants with CTB

**Figure 2:**
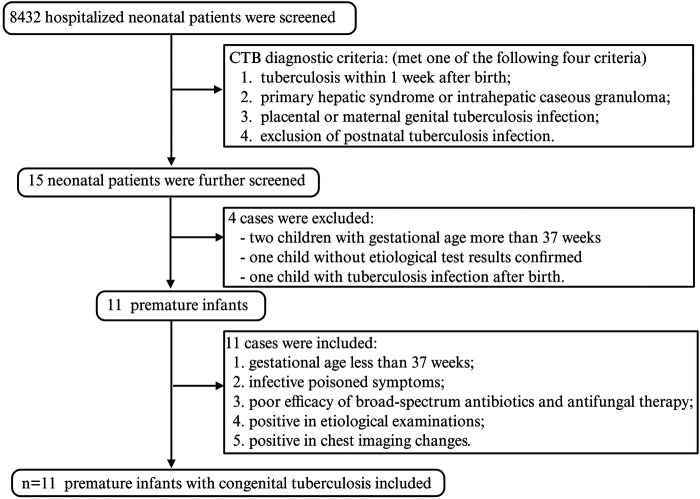
The flowchart of our research. Demographic information and clinical data were obtained from a high-burden, resource-limited NICU in southwest China.

A total of 8,432 hospitalized neonatal patients were involved in the electronic medical records reviewed for this study. Among these neonatal patients, 15 were diagnosed with TB and underwent further screening. Two neonates were excluded because their gestational ages exceeded 37 weeks. One newborn was excluded because of the lack of confirmed etiological results. The other one was not included in the study due to a diagnosis of secondary TB infection after birth. A total of 11 preterm infants were ultimately identified from the hospital's medical records. These infants met the criteria of a gestational age of <37 weeks, negative efficacy of broad-spectrum antibiotics and antifungal therapy, positive symptoms of infectious poisoning, etiological examinations, and abnormal chest images (Figure 2).

Among the 11 preterm infants diagnosed with CTB, the median birth weight was 1,716 g, and the median age of onset was 32 days after birth. The preterm infants with CTB diagnosis included seven males (7/11) and four females (4/11), four natural births (4/11) and seven assisted reproductive techniques (7/11), seven spontaneous births (7/11), and four cesarean sections (4/11). Seven of eleven (7/11) were *in vitro* fertilization and embryo transfer (IVF-ET) recipients and had cesarean sections, experienced fever during the third trimester, were diagnosed with TB, received regular treatment, and had a good prognosis. Four of eleven mothers (4/11) who gave birth naturally were diagnosed with placental or genital TB after their children were delivered. One mother died unexpectedly on the fifth day after giving birth, after developing a fever on the fourth day and having chest CT scans that indicated she had TB, which was awaiting to be confirmed. During the infants’ hospital stay, no household members or medical staff in the NICU had active TB, according to a thorough family contact tracing study. All infants with CTB were preterm, and their mean gestational weeks ranged from 27^+5^ to 34^+2^. Pregnancy complications included diabetes in six of eleven (6/11), placenta previa in two of eleven (2/11), and premature membrane rupture in nine of eleven (9/11), all contributed to their preterm births. All the infants diagnosed with CTB in this research didn't receive Bacillus Calmette–Guérin (BCG) vaccination or tuberculin pure protein derivative (PPD) examination. As for the clinical symptoms at the hospital, the clinical manifestations were very non-specific, mainly characterized by severe infection with toxic symptoms, including loss of appetite in eleven, apnea in ten (10/11), polypnea and cough in nine (9/11), which were the most popular manifestations, followed by low fever and dyspnea in eight (8/11), cyanosis in seven (7/11), jaundice and abdominal distension in five (5/11), and vomiting in three (3/11) preterm infants. In addition, clinical signs included respiratory harshness in nine (9/11), pulmonary moist rales in six (6/11), and hepatomegaly in five (5/11). The number of splenomegaly was relatively small, with only two (2/11) detected (Supplementary File 1).

### 3.2 Blood indicators of patients with CTB at three different time points

Changes in blood examinations were not specific in these 11 patients with CTB, as indicated in Supplementary File 2. Median WBC was 10.92 × 10^9^/L (7.66–30.34), thrombocyte was 253.00 × 10^9^/L (61.00–407.00), percentage of monocyte was 5.85 (5.30–6.30), CRP was 8.05 mg/L (0.05–59.62), and procalcitonin (PCT) was 2.08 ng/mL (0.68–9.22) in the CTB pre-diagnosis group. Median WBC was 16.8 × 10^9^/L (2.9–33.57), thrombocyte was 119.00 × 10^9^/L (64.00–200.00), percentage of monocyte was 7.75 (5.40–10.40), CRP was 69.63 mg/L (38.69–190.00), and PCT was 1.08 ng/mL (0.52–1.87) in the CTB-diagnosed group. Median WBC was 7.71 × 10^9^/L (4.2–20.2), thrombocyte was 271.00 × 10^9^/L (121.00–441.00), percentage of monocyte was 4.04 (3.23–4.70), CRP was 7.07 mg/L(1.38–10.96), and PCT was 0.19 ng/mL (0.06–0.33) in the CTB posttreatment group. Hemoglobin was 120.00 g/L (91.00–142.00) in the pre-diagnosis group, which reduced to 102.00 g/L (86.00–124.00) in the CTB-diagnosed group. For the posttreatment group, median hemoglobin was 147.50 g/L (107.00–200.00), which showed a significant difference compared with that of the CTB-diagnosed group (*P* = 0.001). WBC was lower in the CTB posttreatment group compared with that in the CTB-diagnosed group (*P* = 0.044). Two weeks after receiving anti-TB treatment, thrombocyte count among the patients increased significantly, compared with that of the CTB-diagnosed group (*P* = 0.004). Nevertheless, no significant difference was detected in WBC counts between the CTB pre-diagnosis group and the CTB-diagnosed group (*P* = 0.498). Thrombocytopenia was found in three cases, with an average of 125.40 × 10^9^/L in the CTB-diagnosed group. The percentage of monocytes decreased in the following order: CTB-diagnosed group, CTB pre-diagnosis group, and CTB posttreatment group. In addition, CRP increased significantly when CTB was diagnosed, with a median value of 69.63 mg/L compared with the pre-diagnosis group (*P* = 0.001), and decreased after CTB was treated compared with the posttreatment group (*P* = 0.001). When compared with the pre-diagnosis group, the median PCT was significantly higher in the 11 cases of CTB in the pre-diagnosis group, with a median value of 2.08 ng/mL, lower in the CTB-diagnosed group with a median value of 1.08 ng/mL (*P* = 0.021), and lower in the posttreatment group with a median value of 0.19 ng/mL (*P* = 0.001). Six cases with severe anemia received multiple blood transfusions and oral administration of iron dextran. Thus, anemia was more common in patients diagnosed with CTB and improved following anti-TB and symptomatic treatments (*P* = 0.001). There was no significant difference between the three groups in terms of gender, delivery method, or gestational age at birth of premature babies. Furthermore, red blood cell count, mean corpuscular volume, mean corpuscular hemoglobin, mean corpuscular hemoglobin concentration, percentage of neutrophils and lymphocytes, and red cell distribution width also exhibited no significant differences among the three groups.

Regarding biochemical indicators in serum of these patients with CTB, median total bilirubin was 15.80 mg/dL (7.70–21.0), indirect bilirubin (IB) was 14.36 mg/dL (6.45–20.41), and alanine aminotransferase (ALT) was 15.00 U/L (10–29.00) in the CTB pre-diagnosis group. Median TB was 11.50 mg/dL (7.90–18.20), IB was 10.20 mg/dL (4.70–17.14), and ALT was 32.00 U/L (16.00–147.00) in the CTB-diagnosed group. Median TB was 10.70 mg/dL (7.60–17.60), IB was 9.48 mg/dL (6.00–17.33), and ALT was 23.50 U/L (11.00–176.00) in the CTB posttreatment group. TB levels were significantly decreased in the posttreatment group, compared with those in the pre-diagnosis group (*P* = 0.026). Patients receiving regular anti-TB treatment also had lower IB levels than they did before treatment (*P* = 0.047). ALT levels were significantly higher in CTB-diagnosed patients than those in the pre-diagnosis group (*P* = 0.031). Five of eleven (5/11) had liver function damage following anti-TB treatment, which was resolved by administering compound diisopropylamine dichloroacetate. Only one patient with CTB encountered serious liver damage, whose therapy of rifamycin and isoniazid was suspended for 1 week. This patient kept taking medications even after receiving liver protection treatment. Liver damage caused by factors such as hepatotropic virus infection, parenteral nutrition, and genetic metabolic diseases was ruled out. However, serum levels of biochemical indicators including direct bilirubin, aspartate aminotransferase, total bile acid, and albumin indicated no remarkable differences among the three groups.

Median K level was 4.66 mmol/L (2.75–5.62), median Na level was 136.15 mmol/L (128.50–141.00), median Cl level was 104.70 mmol/L (96.10–114.30), median Ca level was 2.27 mmol/L (2.05–2.84), median P level was 2.07 mmol/L (1.62–2.63), and median Mg level was 0.87 mmol/L (0.69–1.14) in patients pre-diagnosed with CTB. For patients diagnosed with CTB, median K level was 4.32 mmol/L (2.70–5.41), median Na level was 132.15 mmol/L (121.40–139.40), median Cl level was 102.20 mmol/L (93.80–107.70), median Ca level was 1.97 mmol/L (1.24–2.49), median P level was 2.10 mmol/L (1.54–2.57), and median Mg level was 0.65 mmol/L (0.41–1.05). For patients with CTB who have been posttreated with anti-TB medicine, median K level was 4.67 mmol/L (3.71–5.34), median Na level was 136.55 mmol/L (129.6–140.6), median Cl level was 104.85 mmol/L (94.70–110.60), median Ca level was 2.34 mmol/L (2.07–2.65), median P level was 2.18 mmol/L (1.61–2.73), and median Mg level was 0.81 mmol/L (0.68–1.13). Hyponatremia (132.15 mmol/L; 121.40–139.40), hypocalcemia (1.97 mmol/L; 1.24–2.49), and hypomagnesemia (1.97 mmol/L; 1.24–2.49) were more common in the group diagnosed with CTB. There were statistically significant differences in the contents of serum sodium, calcium, and magnesium between the diagnosis group and the pre-diagnosis group (*P* < 0.05) and between the CTB-diagnosed group and the anti-TB posttreated group (*P* < 0.05).

### 3.3 Etiological characteristics of patients with CTB

Sputum AFB smear was positive in four (4/11), gastric juice AFB smear was positive in six (6/11), and no co-infections or other bacterial pathogens were detected in bacterial cultures of these 11 patients with CTB. Considering the possibility of infection, no tuberculous meningitis was found in CSF culturing, and the routine examinations of CSF were negative. MTB liquid culture (BACTEC MGIT) was positive in seven (7/11), and solid culture was positive in five (5/11). Similarly, T-SPOT.TB assays were applied, and five of eleven (5/11) patients with CTB were positive in interferon-gamma release assays (IGRA). Five of eleven (5/11) patients were positive in MTB complex and sensitive to RIF, four (4/11) medium-positive, and two (2/11) lowly-positive in their gastric juice or sputum tested for GeneXpert MTB/RIF. No anti-TB medication resistance was detected (Table 1).

**Table 1:** Characteristics of preterm infants positive for CTB.

Lab number	Sputum TB smear	Gastric juice AFB smear	Other bacterial culturing	CSF routine examination	CSF culturing	Solid culture—LJ	Liquid culture—MGIT	T-SPOT.TB assay	RIF resistance	Xpert MTB/RIF	*C*_t_ value
22082720	Negative	1^+^	Negative	Negative	Negative	Negative	Positive	Positive	Negative	Medium	29.3
22092116	2^+^	2^+^	Negative	Negative	Negative	1^+^	Positive	Positive	Negative	High	17.8
23040201	Negative	Negative	Negative	Negative	Negative	Negative	Negative	Indeterminate	Negative	Low	34.9
22074291	Negative	Negative	Negative	Negative	Negative	2^+^	Positive	Positive	Negative	High	16.3
22040321	2^+^	2^+^	Negative	Negative	Negative	Negative	Negative	Indeterminate	Negative	Medium	28.8
22036483	Negative	1^+^	Negative	Negative	Negative	Negative	Positive	Negative	Negative	Medium	27.6
23049217	1^+^	Negative	Negative	Negative	Negative	1^+^	Positive	Positive	Negative	High	17.4
21048632	Negative	Negative	Negative	Negative	Negative	2^+^	Positive	Positive	Negative	High	17.2
20053265	2^+^	1^+^	Negative	Negative	Negative	Negative	Negative	Indeterminate	Negative	Medium	28.1
19070431	Negative	Negative	Negative	Negative	Negative	Negative	Negative	Negative	Negative	Low	34.5
18062453	Negative	2^+^	Negative	Negative	Negative	1^+^	Positive	Indeterminate	Negative	High	15.8

AFB, acid-fast bacillus; TB, tuberculosis; CSF, congenital tuberculosis; LJ, Lowenstein–Jensen; MGIT, *Mycobacterium* growth indicator tube; RIF, rifampin.

Software on the instrument assigned Xpert MTB/RIF semiquantitative categories. The raw cycle thresholds given in the instrument's output were known as *C*_t_ values.

### 3.4 Imaging and clinical features of patients with CTB

Out of the 11 infants, chest x-ray images were all accessible. Seven of eleven (7/11) were abnormal in x-rays, while four (4/11) were normal. In addition, of the four CTB patients with negative chest x-rays, re-examination of chest CT indicated infiltrated changes. Lung markings were increased in chest CT images; for example, disordered and blurred shadows were common in the 11 positives. Six out of eleven (6/11) patients had enlarged mediastinal lymph nodes, and five out of eleven (5/11) patients had increased foci following broad-spectrum anti-infective treatment. These were progressively absorbed following anti-TB treatment. In addition, the positive changes in chest CT of children with CTB, along with clinical manifestations such as loss of appetite, weight loss, jaundice, low fever, shortness of breath, and dyspnea, are summarized in Table 2. Chest CT abnormalities were observed in all 11 patients with CTB upon re-evaluation. Using a standardized scoring system, the most common findings included bilateral parenchymal involvement in eight out of eleven with a score of 2. Mediastinal lymphadenopathy findings were detected in six of eleven. Atelectasis findings were in five of eleven. None had cavitation or pleural effusion.

**Table 2:** Imaging and clinical features of CTB positives.

Sample number	Lab number	X-ray	Chest CT	Positive imaging changes of chest CT	Clinical characteristics
1	22082720	Positive	Positive	Disordered and blurred shadows, enlarged mediastinal lymph nodes	Appetite loss, apnea, cough, weight loss
2	22092116	Negative	Positive	Disordered and blurred shadows	Appetite loss, apnea, cough, low fever, dyspnea
3	23040201	Positive	Positive	Disordered and blurred shadows, enlarged mediastinal lymph nodes	Appetite loss, jaundice, apnea, cough, weight loss
4	22074291	Positive	Positive	Disordered and blurred shadows	Appetite loss, jaundice, polypnea, low fever, dyspnea
5	22040321	Negative	Positive	Disordered and blurred shadows, enlarged mediastinal lymph nodes, scattered low-density lesions in the occipital lobe	Appetite loss, apnea, low fever, weight loss, dyspnea
6	22036483	Positive	Positive	Disordered and blurred shadows, enlarged mediastinal lymph nodes	Appetite loss, jaundice, apnea, dyspnea, cough
7	23049217	Positive	Positive	Disordered and blurred shadows	Appetite loss, apnea, dyspnea, cough, low fever
8	21048632	Negative	Positive	Disordered and blurred shadows, enlarged mediastinal lymph nodes	Appetite loss, apnea, jaundice, cough, low fever, weight loss, dyspnea
9	20053265	Positive	Positive	Disordered and blurred shadows	Appetite loss, apnea, cough, dyspnea, low fever
10	19070431	Negative	Positive	Disordered and blurred shadows, enlarged mediastinal lymph nodes	Appetite loss, apnea, jaundice, cough, dyspnea, low fever
11	18062453	Positive	Positive	Disordered and blurred shadows	Appetite loss, apnea, cough, low fever

**Figure 3:**
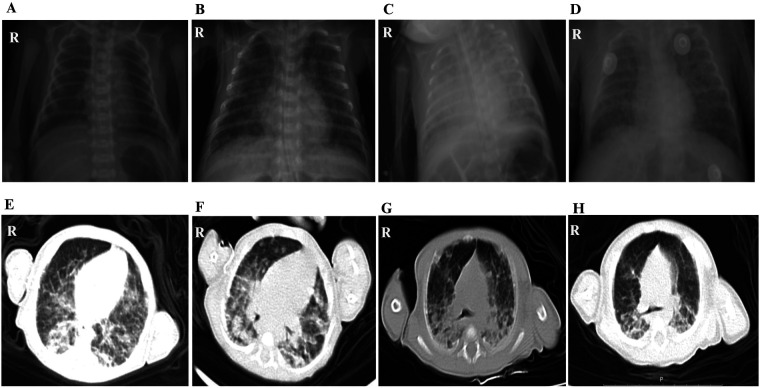
The lung imaging changes of a very premature child of 27^+5^ weeks from birth to diagnosis of CTB to 70 days after treatment. (**A**) Chest radiograph at birth. (**B**) Chest radiograph of CTB was confirmed on the 29th day after birth. (**C**) Chest radiograph on the 38th day after birth, the 9th day after anti-TB treatment. (**D**) Chest radiograph on the 45th day after birth, the 16th day after anti-TB treatment. (**E**) Chest CT scan images on the 57th day after birth, the 28th day after anti-TB treatment. (**F**) Chest CT scan on the 71st day after birth, the 42nd day after anti-TB treatment. (**G**) Chest CT scan on the 85th day after birth, the 56th day after anti-TB treatment. (**H**) Chest CT scan images on the 99th day after birth, the 70th day after anti-TB treatment.

Out of the 11 cases, only one case showed scattered low-density lesions in the occipital and parietal lobes by CT. Although all infants were premature and required respiratory support postnatally, the radiographic imaging before broad-spectrum antibiotics and subsequent response to anti-TB therapy helped distinguish tuberculous lesions from changes related to RDS or BPD. Microbiological confirmation further supported the diagnosis of CTB. Moreover, alterations in lung imaging of a very preterm baby, who was 27^+5^ weeks old at birth, at the time of CTB diagnosis and on the 70th day posttreatment, verified that the child's chest x-ray images at the time of diagnosis showed no obvious signs of primary syndrome or miliary TB. Following standardized anti-TB treatment, the child’s clinical symptoms gradually improved, but the changes in lung imaging still did not improve significantly, and only on the 70th day following anti-TB treatment, lung imaging exhibited the onset of lesion absorption (Figure 3).

### 3.5 Treatment and prognosis

Eight of the eleven infants (8/11) received a combination of intravenous and oral anti-TB therapy and were regularly monitored in our outpatient department. The drug was stopped after a full course of chest imaging with focus absorption for 1 year. The children's healthcare clinic followed up with nine patients with good growth and development. The rest of the two patients discontinued treatment on the 38th and 43rd day and died soon after discharge.

## 4 Discussion

This single-center observation from a high-burden, resource-limited NICU in southwest China highlights factors, including maternal TB detection late in pregnancy and frequent IVF-ET, that may shape presentation and detection workflows, which demand confirmation in multicenter studies. In addition to observing patterns pertinent to endemic, resource-constrained environments in southwest China, it may expand on significant findings from the present literature.

Recent clinical findings support the necessity to trace every child diagnosed with CTB back to a detailed examination of maternal TB history and status (18, 19). The main reason for this tracing is that MTB transmission to the fetus almost always starts with an infected mother (20). Moreover, some reasons make diagnosing active TB during pregnancy extremely difficult, including the high frequency of non-specific TB presentations and the overlap of TB symptoms with typical pregnancy-related symptoms (21). Targeted testing is only possible after the infants have been diagnosed, which makes the pragmatic triage signal extremely difficult for maternal TB. In this study, the mean gestational weeks of the 11 premature infants ranged from 27^+5^ to 34^+2^ weeks. However, mothers who were diagnosed with TB followed by IVF-ET were not screened for TB before pregnancy. This is similar to cases where latent TB reactivation is linked to immunosuppression due to IVF or endometrial damage. Cheng et al. (22) indicated that female disseminated TB is seriously underestimated, and the popularity of assisted reproductive technology may make CTB a major problem. With the widespread use of assisted reproductive technology, more and more infertile women are pregnant with IVF-ET, which can lead to an increase in CTB if the mother does not have a TB assessment before implantation (23, 24). Thus, it may be necessary to improve IVF protocols in TB-endemic regions to incorporate enhanced TB screening before conception, even in asymptomatic women. For example, genital tract imaging, IGRAs assays, and extracellular vesicles of bodily fluids by I-PCR/nanoparticle-based I-PCR are suggested to be taken into account (25, 26). If genital TB is diagnosed, it is recommended to complete treatment first before attempting to conceive.

The inherent diagnostic difficulty in CTB is largely due to the profound lack of specificity in its routine measurement of inflammatory biomarkers. According to earlier research, acute-phase reactants and hematological abnormalities, such as elevated CRPs, abnormal WBC counts (leukocytosis or a left shift), thrombocytopenia, variable PCT elevations, hypocalcemia, and hypomagnesemia were all indicative of systemic inflammatory responses in infants with CTB (27, 28). Critically, the biomarkers observed in severe neonatal bacterial or fungal sepsis were strikingly similar to the abnormalities of blood laboratory indicators, making early diagnosis of CTB extremely difficult. In this study, a significant increase in CRP during the active period was consistent with its function as an acute-phase reactant, representing systemic inflammation triggered by the mycobacterial burden. In addition, the regular anti-TB normalization of CRP posttreatment reconfirmed its usefulness for therapeutic monitoring. CRP was an acute-phase reactant produced in response to pro-inflammatory cytokines such as IL-6, which were elevated in active TB (29). In patients with CTB, their disseminated MTB infection triggered a systemic inflammatory response, leading to elevated CRP. Its normalization posttreatment reflects reduced bacterial burden and inflammatory activity. On the contrary, PCT demonstrated an unexpected reduction during active disease, contradicting its typical feature in bacterial sepsis. The reason for this paradox was probably that MTB had the unusual ability to inhibit PCT synthesis through immunomodulatory mechanisms of FcRs binding profiles and glycosylation patterns, according to a recent pediatric TB study (30).

Anemia was also more frequently observed in preterm infants with CTB within our cohort study. This finding was consistent with the pathophysiological mechanisms of TB in the previous studies. For example, anemia of chronic disease, which resulted from ongoing systemic inflammation, was a well-known TB symptom (31). Pro-inflammatory cytokines such as tumor necrosis factor-α (TNF-α) and interleukin-6 (IL-6) elevated the expression of hepatic hepcidin, which limited the amount of iron available for erythropoiesis by preventing intestinal absorption and trapping iron inside macrophages (32, 33). Red blood cell production was impaired, and functional iron deficiency resulted from this inflammatory blockade. In addition, bone marrow suppression induced by mycobacterial infection and cytokine dysregulation may weaken erythroid progenitor proliferation, further leading to anemia (34). Furthermore, infants with CTB often presented with nutritional compromise, low birth weight, and prematurity, which exacerbated risks of iron-deficiency anemia in return (35). Chronic infections in the early neonatal stage might increase metabolic demand and damage erythropoiesis. Finally, hepatosplenomegaly often coexisted with CTB, which might result in hypersplenism and promote peripheral destruction of red blood cells (36, 37). Taken together, these mechanisms might explain the phenomenon of anemia is more common among preterm infants with CTB, compared with the others. Clinically, anemia recognition in this situation might indicate risks of CTB, particularly in high-burden TB regions. Severe thrombocytopenia during active CTB was also associated with disseminated disease. It is consistent with the consumptive coagulopathy that Peng et al. (27) verified 41% of infants with disseminated TB, where fatal outcomes were predicted by platelet counts <100 × 10⁹/L (OR = 3.8). Furthermore, the platelet recovery posttreatment may verify the evidence of MTB removal. Thrombocytopenia in disseminated TB might result from consumptive coagulopathy, bone marrow suppression, or immune-mediated platelet destruction (38). In neonates, immature immune responses and disseminated infection may exacerbate this phenomenon. According to recent pharmacovigilance reports, preterm neonates' immature cytochrome P450 systems increased their risk of hepatotoxicity (39, 40). Our study indicated that less than half patients developed ALT elevation after treatment, which was directly linked to liver damage caused by first-line anti-TB medications. Notably, hyperbilirubinemia resolved faster than ALT elevation posttreatment, also suggesting mechanisms of drug-induced hepatocyte injury, rather than other reasons for hemolytic anemia. Consistent with previous studies, the three symptoms of hyponatremia, hypocalcemia, and hypomagnesemia during active TB indicated multiple-organ dysfunctions (41–43). The group with CTB also had higher rates of hyponatremia, hypocalcemia, and hypomagnesemia in our study. Hyponatremia might result from syndrome of inappropriate antidiuretic hormone secretion (SIADH) due to central nervous system involvement or systemic inflammation (44). Hypocalcemia and hypomagnesemia might occur due to malabsorption, renal tubular dysfunction, or cytokine-mediated disturbances in mineral metabolism. TB-associated malnutrition and prematurity may exacerbate these deficiencies (45). In addition, the framework of immunological immaturity linked to prematurity needs to be taken into consideration when interpreting hematological and inflammatory parameters in this preterm infant cohort with CTB. The gestational age of the infants in our study ranged from very preterm to late preterm, a period during which innate and adaptive immune functions are developmentally compromised. Therefore, while significant fluctuations in parameters such as WBC count, thrombocytes, monocytes, and CRP in relation to CTB diagnosis and treatment were observed, these changes occurred against a baseline already altered by prematurity.

Prior study confirmed that MTB maintained bacillary integrity for >4 h after ingestion by resisting gastric acidity through thick, lipid-rich cell walls and intra-macrophagic survival (46). The positive rate of AFB smear in body fluid was high, especially in gastric juice, with the advantages of ease to collect samples, less trauma, and low detection cost, which made it to be adopted in early screening (47, 48). Our gastric aspirate AFB smear positivity was lower than the 80% reported. Several factors were likely to contribute. First of all, our cohort comprised exclusively preterm infants with CTB in a high-burden, resource-limited NICU, with a phenotype skewed toward extrapulmonary involvement, for example, mediastinal lymphadenopathy in six out of eleven, and normal chest radiographs in four. These features may reduce endobronchial bacillary shedding and gastric bacillary load. Moreover, our testing occurred at a median of 32 days, which was later than many perinatal TB cases. Thus, bacillary dynamics in late-presenting, nodal-predominant CTB may be less favorable for smear detection. Furthermore, our sample size was limited to 11 patients, whose estimate was imprecise, with approximately 28%–79% of the 95% CI. Our sampling variability may account for part of the observed difference. Importantly, MGIT culture and Xpert MTB/RIF positivity were relatively high in our cohort, reinforcing that molecular and culture-based methods are critical complements to smear microscopy in preterm infants with CTB. These findings were consistent with previous reports of neonatal anatomy and MTB pathophysiology. The combined gastric juice Xpert detection and blood T-Spot detection could increase the diagnosis rate of CTB. The diagnosis rate of CTB may be increased by combining blood T-Spot detection with gastric juice Xpert. As previously reported, the sensitivity of PCR in diagnosing CTB was 60%, and the specificity was 97% (49). Almost all patients were positive for gastric juice Xpert MTB/RIF, which could be considered as positive evidence of CTB.

The clinical manifestations of CTB were atypical. The most common symptoms include low fever, feeding difficulties, cough, irritability, dysplasia, weight loss, respiratory distress, hepatosplenomegaly, enlarged lymph nodes, and abdominal distension (50). In the early stage, preterm infants were admitted to the hospital primarily because of poor reaction and shortness of breath after preterm delivery. After routine anti-infectious treatment, the condition of the premature infants improved. They then exhibited different extents of less eating, apnea, polypnea, cough, low fever, dyspnea, cyanosis, jaundice, abdominal distension, and vomiting. The above clinical manifestations were extremely difficult to distinguish from infectious pneumonia or neonatal sepsis, especially in ultra-premature infants during hospitalization, who were severely infected and had poisoning symptoms, with negative results posttreatment with broad-spectrum antibiotics (51, 52).

As for the chest CT scan image, changes of CTB were not very specific. In this study, pulmonary imaging findings of 11 infants indicated increased lung texture, disorder and blur, patches, no typical primary syndrome, and miliary pulmonary TB. Some patients showed mediastinal lymph node enlargement, with no obvious hilar lymph node enlargement. Nevertheless, the patchy shadow also had its characteristic: the pulmonary lesions worsened gradually before diagnosis, showed no improvement after broad-spectrum antibiotic treatment, and the lesions were gradually absorbed after anti-TB treatment. There was no hepatosplenomegaly reported in this group. As reported in the cranial CT imaging of this infant revealed scattered low-density lesions in the occipital and parietal lobes. This was the only one in our cohort with documented central nervous system (CNS) lesions. His general clinical presentation was poor activity, apnea, feeding difficulties, and respiratory distress. In the context of CTB, neurological symptoms might be subtle and overlap with sepsis or prematurity-related complications, making clinical distinction challenging. No tuberculous meningitis was found in CSF culturing, and the routine examinations of CSF were negative. This was not rare in early or localized CNS TB without meningeal involvement. Isolated parenchymal tuberculomas may not shed bacilli into the CSF, especially in early stages. The absence of CSF abnormalities implied that an immature blood–brain barrier in preterm infants may facilitate bacillary entry, but the immune response may be attenuated, delaying typical CSF changes. Thus, the current case highlighted the significance of taking neuroimaging into account in infants with CTB who have persistent or inexplicable symptoms, even when their CSF is normal.

**Figure 4:**
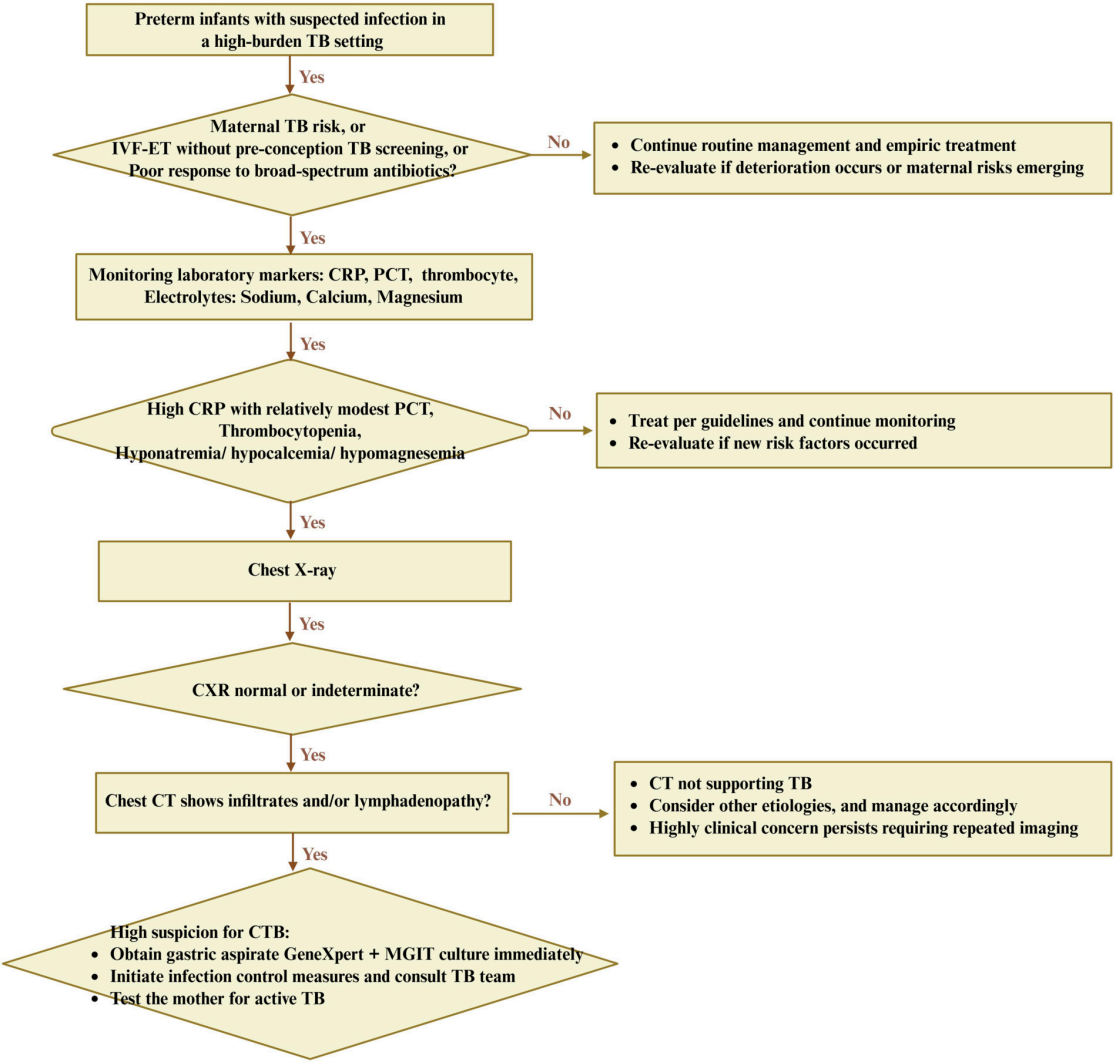
Hypothesis-generating diagnostic flowchart for CTB in preterm infants from a high-burden TB setting. A triage bundle mainly represented clinical contexts, laboratory markers, and pulmonary imaging discordance. Clinical contexts primarily included maternal TB risk, IVF-ET without pre-conception screening, or poor response to broad-spectrum antibiotics. A reversible laboratory marker included high CRP with relatively modest PCT, thrombocytopenia, and concurrent hypo-sodium/calcium/magnesium. Image discordance usually represented as normal or indeterminate x-ray with CT abnormalities, which deserve early gastric aspirate GeneXpert plus MGIT culture, infection control, and maternal evaluation. This flowchart is hypothesis-generated and requires validation in controlled cohorts. CXR, chest x-ray. IVF-ET, *in vitro* fertilization and embryo transfer; MGIT, *Mycobacterium* growth indicator tube.

Pragmatic triage and timely treatment can improve the survival rates of CTB. Nine patients with CTB survived after treatment, and two patients died with poor prognosis. Combined with the present observing patterns of this group with CTB, it is suggested that mothers with a history of infertility in areas with high incidence of TB should pay attention to rule out the possibility of active TB after assisted reproductive assistance, to promote the triage and treatment of CTB (53). For this research, the experiences were obtained as follows. First, for premature infants in high-risk areas for TB, it is suggested to focus on the early examination of CTB, repeatedly ask about the causes of preterm delivery, complete the pathological examination of the mother's placenta and fetal membrane tissue as far as possible, look for the causes of premature delivery, and pay more attention to the mothers with assisted reproduction and repeated abortion. Second, during hospitalization, constant observation of disease changes is important. Premature infants with similar sepsis should be actively checked for TB infection from multiple aspects, and pragmatic triage should be carried out. For infants whose mothers have been diagnosed with TB infection, it is necessary to search for signs of TB infection early, repeatedly, and begin timely treatment. Fourth, joint obstetrics and infection departments for prenatal education should be recommended; e.g., women with a history of infertility should undergo improved TB screening, especially in the case of reproductive TB, to move the treatment window forward so the family can have a better chance of a healthy baby (Figure 4). Finally, for confirmed cases, it is suggested to adhere to standardized treatment to improve the prognosis.

In addition, there are a number of limitations in this study that should be considered. For example, our study is a single-center, cross-sectional study with a small sample size, a lack of contemporaneous non-CTB preterm controls, potential selection bias, and unmeasured confounders. Thus, our findings are intended to guide clinical suspicion and workflows, and not to establish definitive diagnostic criteria or the regional uniqueness of CTB. We acknowledge that in the high-burden, resource-limited settings of southwest China, genotyping to confirm strain identity between mother and infants is not always accessible. However, the combination of clinical, epidemiological, and laboratory data may provide strong evidence for CTB. Future research is recommended to conduct a multicenter, matched case–control design that includes preterm infants with CTB, gestation age/birth weight-matched non-CTB, and a pre-registered protocol to validate the triage bundle and estimate diagnostic accuracy.

## 5 Conclusions

This cross-sectional study indicated that effective triage of CTB in premature infants in southwest China was extremely difficult. The symptoms of infants with CTB were atypical, which induced a late onset time. In high-incidence areas of TB, early detection of CTB in premature infants with laboratory abnormalities (anemia, thrombocytopenia, hyponatremia, hypocalcemia, hypomagnesemia) requires nucleic acid amplification testing furtherly. Infants born via assisted reproduction or whose mothers were exposed to TB should pay particular attention. Patients with CTB under standardized anti-TB therapy required constant hepatotoxicity monitoring.

## Data Availability

The raw data supporting the conclusions of this article will be made available by the authors, without undue reservation.
